# Влияние хирургического лечения морбидного ожирения на заболевания суставов

**DOI:** 10.14341/probl13258

**Published:** 2023-08-30

**Authors:** Т. С. Паневин, Е. Г. Зоткин, А. М. Лила

**Affiliations:** Научно-исследовательский институт ревматологии им. В.А. Насоновой; Научно-исследовательский институт ревматологии им. В.А. Насоновой; Научно-исследовательский институт ревматологии им. В.А. Насоновой; Российская медицинская академия непрерывного профессионального образования

**Keywords:** ожирение, бариатрическая хирургия, ревматоидный артрит, подагра, остеоартрит, псориатический артрит

## Abstract

Распространенность ожирения в современном мире возрастает. Ожирение является независимым фактором риска некоторых ревматических заболеваний, а также ухудшает их течение. Наличие хронического заболевания суставов может представлять трудность для лечения ожирения в плане уменьшения активности, создавая своеобразный порочный круг, когда суставные боли затрудняют выполнение физических нагрузок, а избыток массы тела усиливает боли в суставах. В то же время существует условно радикальный метод лечения ожирения — бариатрическая хирургия (БХ), который на сегодняшний день применяется при неэффективности консервативных методов. Целью настоящего обзора является анализ представленных в мировой литературе данных о влиянии БХ на течение наиболее распространенных ревматических заболеваний. Имеющиеся данные показывают возможность положительного влияния БХ не только на снижение массы тела, но и на течение ряда ревматических заболеваний.

## Abstract (en)

The prevalence of obesity in the modern world is increasing. Obesity is an independent risk factor for some rheumatic diseases and also worsens their course. The presence of chronic joint disease can make it difficult for obesity to reduce activity, creating a vicious circle where joint pain makes exercise difficult and being overweight exacerbates joint pain. At the same time, there is a conditionally radical method of treating obesity — bariatric surgery (BS), which is currently used when conservative methods are ineffective. The purpose of this review is to analyze the data presented in the world literature on the impact of BS on the course of the most common rheumatic diseases. The available data show the possibility of a positive effect of BS not only on weight loss, but also on the course of a number of rheumatic diseases.

## ВВЕДЕНИЕ

Распространенность ожирения в современном мире возрастает [1]. Ожирение является независимым фактором риска некоторых ревматических заболеваний, а также ухудшает их течение [2]. Вместе с тем существует ряд трудностей в лечении ожирения. Поскольку заболевание обусловлено внешнесредовыми и генетическими факторами, важное значение имеет образ жизни. В регуляции аппетита значимую роль играет как обмен нейромедиаторов, так и гормоны инкретины, синтезируемые нейроэндокринными клетками желудочно-кишечного тракта [3]. В силу гетерогенности патогенетических факторов, тенденции к нарастанию малоподвижного образа жизни и увеличению хронического стресса, а также относительную скудность представленного на территории РФ выбора медикаментозной терапии ожирения, длительно поддерживаемое устойчивое достижение нормальной массы тела затруднено [4]. Только около 10% снизивших массу тела пациентов могут добиться поддержания нормальной массы тела в течение длительного времени [5].

Наличие хронического заболевания суставов представляет определенную трудность для лечения ожирения в плане уменьшения активности, создавая, таким образом, своеобразный порочный круг, когда суставные боли затрудняют физическую нагрузку, что способствует набору массы тела, а избыточная масса тела как за счет своего прямого «механического» влияния, так и повышения синтеза провоспалительных цитокинов (в первую очередь интерлейкина-6 и фактора некроза опухолей альфа (ФНО-α)) может усугублять суставной синдром [2].

В то же время существует условно радикальный метод лечения ожирения — бариатрическая хирургия (БХ), который на сегодняшний день применяется при неэффективности немедикаментозного и медикаментозного лечения у пациентов с индексом массы тела более 40 кг/м² или более 35 кг/м² при наличии сопутствующих заболеваний, течение которых может улучшаться после проведения бариатрического оперативного вмешательства, — например, сахарного диабета 2 типа, дислипидемии, синдром ночного апноэ и т. д. [3]. В настоящее время для хирургического лечения ожирения могут быть проведены временная эндоскопическая установка внутрижелудного баллона, регулируемое бандажирование желудка, гастропликация, рукавная резекция желудка, гастрэктомия с формированием анастомоза по Ру, а также рукавная резекция желудка, дополненная билио-панкреатическим шунтированием. Современные оперативные технологии позволяют выполнять большинство вышеуказанных оперативных вмешательств лапароскопическим путем, что уменьшает число послеоперационных осложнений и расширяет круг пациентов, которым может быть выполнена БХ. Целью настоящего обзора является анализ представленных в мировой литературе данных о влиянии БХ на течение наиболее распространенных ревматических заболеваний.

## ВЛИЯНИЕ БАРИАТРИЧЕСКОЙ ХИРУРГИИ НА РЕВМАТИЧЕСКИЕ ЗАБОЛЕВАНИЯ

## Ревматоидный артрит.

Недавний метаанализ 11 исследований показал связь между индексом массы тела (ИМТ) и риском развития ревматоидного артрита (РА): пациенты с ожирением имели на 24% более высокий риск РА по сравнению с группой лиц с нормальным ИМТ [6]. Наличие ожирения при раннем РА снижает шансы достижения ремиссии на 43% и поддержания ремиссии на 51% [7]. Пациенты с ожирением имеют более высокие показатели активности РА, менее выраженную положительную реакцию на лечение (в первую очередь ингибиторы ФНО) [8], более низкое качество жизни и более интенсивную суставную боль [9], но в то же время меньшую склонность к формированию суставных эрозий, что может быть обусловлено протективным действием адипонектина [10]. По данным исследования SOS (Swedish Obese Subjects Study), у пациентов с ожирением проведение БХ не являлось фактором риска развития РА [11]. Потеря веса за счет изменения образа жизни и диеты уменьшает клинические проявления РА [12], однако, по данным других исследований, снижение массы тела при РА ассоциируется с большей потерей трудоспособности [13], что может быть связано со снижением веса на фоне активности и прогрессирования РА. Кроме того, ожирение ассоциировано с классическими факторами риска сердечно-сосудистых заболеваний, значимо увеличивая 10-летний риск кардиоваскулярных событий у пациентов с РА [14].

В ретроспективном исследовании 53 пациентов с РА, которым выполнялись различные бариатрические вмешательства (гастрошунтирование, рукавная резекция желудка и регулируемое бандажирование), через 12 мес отмечалось снижение числа пациентов с умеренной/высокой активностью с 57% до 6% (р<0,001), а также снижение острофазовых показателей (снижение уровня С-реактивного белка (СРБ) с 26,1±20,9 до 5,9±8,2 мг/л; p<0,001; снижение СОЭ с 45,7±26,2 до 26,1±2,0 мм/ч; p<0,05) [15].

В проспективном когортном исследовании пациентов с РА после БХ (n=32) отмечено улучшение индексов ACR20, ACR50 и ACR70 по сравнению с контрольной группой (n=33), получавшей только медикаментозную терапию РА (p<0,05), а также более низкие значения индексов DAS28 СОЭ и DAS28 СРБ через 12 мес 1,5±0,9 vs 2,4±1,4 и 1,2±0,9 vs 2,2±1,7 соответственно (p<0,05) [16].

В 2020–2021 гг. были сформулированы основные положения о тактике ведения пациентов с так называемым трудно-поддающимся лечению ревматоидным артритом (difficult to treat rheumatoid arthritis — D2T RA) [17][18]. В качестве одной из точек зрения с уровнем доказательности С и высоким (9,2 из 10) уровнем согласия экспертов является то, что наличие ожирения может негативно влиять на значение показателя активности РА, что, вероятно, может быть обусловлено как прямым повышением уровня СРБ или СОЭ, так и вышеописанными провоспалительными механизмами. Вопрос влияния медикаментозной и хирургической терапии ожирения, в том числе с целью преодоления резистентности к противовоспалительной терапии, требует дальнейшего изучения посредством проспективных исследований.

## Остеоартрит (ОА).

Ожирение является независимым фактором риска ОА [19]. Пациенты с ИМТ>30 кг/м2 в 7 раз чаще страдают ОА по сравнению с пациентами с индексом массы тела <25 кг/м² [20]. Следует отметить, что увеличение веса является фактором риска не только ОА коленных суставов, но и суставов верхних конечностей, для которых сам по себе избыток массы тела не является столь очевидным фактором риска [21], что связывают с увеличением синтеза провоспалительных цитокинов.

Результаты имеющихся исследований показывают в целом положительное влияние БХ на течение ОА крупных суставов нижних конечностей. Так, при проспективном изучении 24 пациентов с ОА коленного сустава, которым была выполнена БХ со средней потерей веса 27% за 2 года, отмечено улучшение по шкалам KOOS и WOMAC через 6, 12 и 24 мес [22]. В исследовании 44 пациентов с умеренным/тяжелым ОА коленного сустава потеря веса сопровождалась снижением уровней ИЛ-6, CРБ, фибриногена и орозомукоида, а также увеличением N-концевого пропептида коллагена IIA (+32%) и снижением хрящевого олигомерного матриксного белка (COMP; –36%) [23]. В еще одном исследовании продемонстрировано увеличение суставной щели на ~0,7 мм у 64 пациентов по данным рентгенографии коленного сустава на фоне снижения ИМТ на 6,3 кг/м² через 3 мес [24].

Возрастает и число пациентов с ожирением, которым проводится тотальное эндопротезирование (ТЭП) крупных суставов по поводу ОА [25]. У больных с ожирением чаще встречаются послеоперационные осложнения после проведения артропластики [26]. Несмотря на увеличение подвижности суставов после ТЭП, большинство пациентов сохраняют или продолжают набирать избыточную массу тела [27]. Во многих исследованиях показан положительный эффект БХ, предшествующей проведению ТЭП. При этом вопрос наиболее оптимального интервала между двумя вышеуказанными оперативными вмешательствами остается дискуссионным: существует точка зрения о проведении ТЭП после достижения устойчивых цифр веса на фоне похудения после БХ.

В ретроспективном исследовании среди пациентов с морбидным ожирением (n=2636 в каждой группе), которым было выполнено ТЭП коленного сустава, среди тех, кому предварительно была выполнена БХ, встречалось меньше внутригоспитальных осложнений в течение последних 90 дней [28]. В другом исследовании отмечено снижение частоты повторных вмешательств по поводу ТЭП тазобедренного сустава при предварительном проведении БХ у пациентов с ожирением [29]. В еще одном исследовании пациенты с морбидным ожирением и БХ до ТЭП имели в 3,5 раза меньший риск раневой инфекции и в 7 раз меньший риск повторной госпитализации [30]. Отмечено снижение числа дней госпитализации по поводу артропластики у тех пациентов с ожирением, кто ранее перенес БХ при схожем показателе ранних послеоперационных осложнений и необходимости повторных вмешательств [31].

Таким образом, БХ может быть эффективным вспомогательным методом коррекции факторов риска прогрессирования ОА у лиц с выраженным ожирением. Важными перспективами изучения в данной области могут быть исследования, оценивающие динамику клинико-рентгенологических показателей при различных фенотипах ОА, а также влияние БХ на возможное уменьшение риска замены суставов.

## Подагра.

Подагра является аутовоспалительным заболеванием, важную роль в патогенезе которого играют нарушение пуринового обмена и стойкое повышение уровня мочевой кислоты (МК) — гиперурикемия (ГУ), что приводит к образованию кристаллов моноурата натрия. Важность изучения бариатрической хирургии для данных пациентов обусловлена высокой распространенностью ожирения при подагре, возможностью влияния бариатрической хирургии на метаболические показатели, в том числе на уровень МК, а также тот факт, что любое хирургическое вмешательство является фактором риска обострения подагры [32].

Метаанализ 20 исследований влияния БХ на уровень МК и подагру, большинство (n=14) из которых продолжалось более одного года со средним исходным показателем ИМТ перед операцией 45,2 кг/м², показал средний предоперационный уровень МК 6,5 мг/дл. Отмечено снижение уровня МК в среднем на 0,73 мг/дл с 3-го месяца после операции, которое сохранялось и на третий год после операции (-1,91 мг/дл). Большее снижение ИМТ положительно коррелировало со снижением уровня МК. Значимое снижение частоты приступов подагры также было продемонстрировано с 3-го месяца наблюдения (0–35 % до БХ и 7–19% — через 3 мес после проведения) [33].

В исследовании N. Dalbeth и соавт. (2014 г.) 60 пациентам с СД2 и ИМТ≥35 кг/м², 12 из которых страдали подагрой, была проведена лапароскопическая рукавная резекция желудка с последующим наблюдением в течение 1 года, за который средняя потеря массы тела составила 34,3 (±11,1) кг [34]. Уровень МК выше 360 мкмоль/л отмечался в 10 из 12 (83%) случаев подагры до оперативного вмешательства и в 4 из 12 случаев (33%) через год после операции. Интересно отметить, что потребность в применении уратснижающей терапии уменьшилась с 75% (9 из 12 пациентов) до 33% (4 из 12 пациентов). Данный факт может иметь важное значение, поскольку подагра является ревматическим заболеванием с наиболее выраженным метаболическим компонентом, а основная цель лечения заболевания — длительное поддержание целевого уровня МК, и возможно предполагать, что БХ может быть методом предотвращения новых приступов.

Метаанализ 10 исследований влияния снижения веса на уровень МК и течение подагры показал, что общее снижение веса на >7 кг суммарно и/или >2 кг в неделю любым способом (хирургически или консервативно) имело положительный эффект на уровень МК в средне- и долгосрочной перспективе [35]. От 0 до 60% пациентов достигли нормального значения уровня МК (<360 мкмоль/л). В 6 из 8 исследований (75%) был установлен положительный эффект снижения массы тела на течение подагры. Снижение веса на >3,5 кг суммарно показало положительный эффект на снижение числа приступов подагры в средне- и долгосрочной перспективе. Кроме того, отмечено повышение уровня МК и числа обострений подагры в раннем послеоперационном периоде.

Как упоминалось выше, хирургическое вмешательство является фактором риска обострения подагры. Это может быть связано с относительным обезвоживанием в рамках предоперационной подготовки, длительным голоданием, а также отменой противовоспалительной и уратснижающей терапии перед оперативным лечением [32]. В сравнительном исследовании Romero-Talamás H. и соавт. [36] 155 пациентов с подагрой, 99 из которых была выполнена БХ и 56 пациентов, которым были выполнены небариатрические оперативные вмешательства, отмечена более высокая частота приступов после БХ в 1-й месяц после оперативного лечения (17,5% vs 1,8%, p=0,003), в то время как через один год отмечено снижение частоты обострений с 23,8% до 8,0% в группе БХ (p=0,005), что сопровождалось значимым снижением уровня МК (c 9,1±2,0 до 5,6±2,5 мг/дл, p=0,007), при отсутствии схожей значимой динамики в группе небариатрических вмешательств (с 18,2% до 11,1%, p=0,33). Факторами более высокой частоты приступов при БХ в послеоперационном периоде могут быть ускоренный катаболизм вследствие стремительной потери веса, массивное разрушение тканей желудка, а также высокобелковая диета.

Динамика влияния бариатрических операций на уровень МК показана в исследовании Xu C. и соавт. [37]. В исследование были включены 165 пациентов с ГУ, которым были выполнены различные виды бариатрических вмешательств. 103 (62,4%) пациентам выполнена лапароскопическая рукавная гастрэктомия (LSG), 54 (32,7%) — рукавная гастрэктомия с формированием еюно-еюнального анастомоза (SG + JJB), еще 8 (4,8%) — лапароскопическая гастрэктомия с формированием анастомоза по Ру (LRYGB). Средний уровень МК значимо снижался с 489,4±93,7 до 372,6±101,4 мкмоль/л через сутки после операции, а затем повышался до 531,6±175,5 мкмоль/л через 1 мес наблюдения с последующим снижением до 415,2±105,6 и 396,5±114,2 мкмоль/л через 3 и 6 соответственно.

Таким образом, бариатрические вмешательства сопровождаются транзиторным повышением уровня МК в раннем послеоперационном периоде, что в совокупности с другими вышеописанными факторами может приводить к развитию острого подагрического приступа. Однако в последующем послеоперационное уменьшение веса сопровождается устойчивым снижением уровня МК и вероятности обострения подагры в отдаленном периоде, снижению дозы уратснижающих препаратов, а в некоторых случаях и их полной отменой.

## Псориатический артрит.

Ожирение является фактором риска псориаза и псориатического артрита (ПсА) [38]. Пациенты с псориазом и сопутствующим ожирением хуже отвечают на лечение, в то же время низкокалорийная диета ассоциируется с улучшением течения псориаза (его распространенности, тяжести и качества жизни) [39].

Ретроспективный анализ 1991 пациентов с ожирением из шведского исследования SOS (Swedish Obese Subjects Study) показал, что скорректированное отношение рисков (сОР) развития псориаза уменьшалось при проведении БХ до 0,65 (95% ДИ 0,47–0,89, р=0,008), в то время как для ПсА данное снижение было незначимым (сОР 0,71; 95% ДИ 0,38–1,33; р=0,287). Все разновидности проводимых операций (вертикальная гастропластика, бандажирование желудка и гастрошунтирование) были эффективны в плане снижения риска развития псориаза [40].

В ретроспективном анализе среди 86 больных псориазом, 21 из которых страдали ПсА [41], после проведения бариатрического вмешательства показано значимое снижение активности псориаза и ПсА по шкале Disease severity rating scale (0–10) с 5,6 до 4,4 балла (p<0,01) и с 6,6 до 4,5 баллов (p<0,01).

Датское когортное исследование пациентов с БХ, проводимое в 1997–2012 гг., показало, что среди пациентов, перенесших гастрошунтирование (n=12 364), отмечалось снижение риска развития псориаза (сОР 0,52 (95% ДИ 0,33–0,81)), «утяжеления» псориаза (сОР 0,44 (95% ДИ 0,23–0,86)), а также риска развития ПсА (сОР 0,29 (95% ДИ 0,12–0,71)), в то время как для группы регулируемого бандажирования желудка (n=1071) по всем трем критериям значимого уменьшения получено не было [42]. Одна из возможных причин данных результатов — различное влияние на уровень ГПП-1 вышеуказанных оперативных методик. Регулируемое бандажирование создает уменьшение объема желудка, в то время как анатомические изменения желудочно-кишечного тракта после гастрошунтирования сопровождаются изменениями уровня инкретинов.

Представленные исследования косвенно свидетельствуют о наличии положительного эффекта БХ на течение ПсА. Учитывая частое сочетание ПсА с различными метаболическими нарушениями, необходимо проведение дальнейших проспективных исследований в данном направлении.

## ВОЗМОЖНЫЕ НЕГАТИВНЫЕ ЭФФЕКТЫ БАРИАТРИЧЕСКОЙ ХИРУРГИИ В КОНТЕКСТЕ РЕВМАТИЧЕСКИХ ЗАБОЛЕВАНИЙ

Помимо риска обострения острого артрита у пациентов с подагрой, существует еще ряд негативных эффектов и трудностей, с которыми могут столкнуться пациенты после проведения БХ. К их числу можно отнести нарушение обмена кальция и витамина D с развитием остеопороза, артропатию, а также фармакокинетические проблемы при применении традиционной противовоспалительной терапии.

Предоперационный дефицит витамина D наблюдается у 60–70% пациентов с ожирением [43]. Поскольку всасывание витамина D происходит в тощей и подвздошной кишке, оперативные вмешательства, затрагивающие данные отделы, такие как гастро- или билиопанкреатическое шунтирование, могут приводить к нарушению его метаболизма. Эти же виды вмешательств влияют и на усвоение кальция, что сопровождается компенсаторным повышением уровня паратгормона (ПТГ) и снижением минеральной плотности костной ткани (МПКТ), в то время как регулируемое бандажирование и рукавная резекция желудка вызывают минимальные изменения фосфорно-кальциевого обмена. Еще одним фактором снижения МПКТ может быть снижение уровня эстрогенов, сопровождающее уменьшение избыточного количества жировой ткани, в которой продуцируется эстрон. Изменения уровня адипокинов и гастроинтестинальных гормонов после БХ также могут иметь значение, поскольку они влияют на метаболизм костной ткани [44].

Исследования, изучавшие изменения МПКТ у пациентов после гастрошунтирования, показали ее снижение в проксимальном отделе бедра примерно на 8–10% без явного снижения МПКТ в поясничном отделе, а также повышение маркера остеорезорбции N-телопептида коллагена 1 типа в моче, повышение уровня ПТГ, несмотря на прием препаратов кальция и витамина D [45][46]. Значимыми факторами снижения МПКТ в данных исследованиях были постменопаузальный статус и выраженность потери веса.

Исследование Vilarrasa N. и соавт., сравнивающее влияние рукавной резекции и гастрошунтирования на МПКТ, не выявило разницы в проценте пациентов с остеопенией или остеопорозом между 2 группами через 1 год после оперативного вмешательства, при этом постменопаузальный статус также был ассоциирован с большим риском снижения МПКТ [47]. В целом, несмотря на снижение МПКТ, наблюдаемое в данных исследованиях, значения МПКТ часто остаются в пределах нормы [48].

Данные о влиянии БХ непосредственно на риск остеопоротических переломов противоречивы. Lalmohamed A. и соавт. не обнаружили повышения частоты остеопоротических переломов у пациентов через 2,2 года после бариатрической операции по сравнению с контрольной группой, хотя наблюдалась тенденция к увеличению риска переломов через 3–5 лет после операции и у пациентов с более высокой потерей веса [49]. По данным обследования 258 жителей США, наблюдаемых в среднем в течение 7,7 года после БХ, было показано 2-кратное увеличение риска традиционных остеопоротических переломов [50].

С учетом этих данных, рекомендуется [51] проводить ежегодный скрининг уровней ПТГ, 25(ОН)витамина D, альбумина и кальция в крови, суточной экскреции кальция с мочой. Рекомендованное суточное потребление кальция варьирует от 1500 мг/сут до 2400 мг/сут в зависимости от выбора вмешательства, лучше использовать цитрат кальция из-за меньшего влияния концентрации желудочного сока на его всасываемость. В раннем послеоперационном периоде может потребоваться не менее 3000 МЕ витамина D.

Разновидности бариатрических операций, выполняемых в 1980-х годах, ассоциировались с развитием послеоперационного неэрозивного серонегативного артрита, что, вероятнее всего, было обусловлено тощекишечно-подвздошным шунтированием, приводящим к длительному нахождению микробиоты в просвете кишечника и антигенной стимуляции [52], что подтверждается купированием проявлений артрита после устранения анастомоза [53].

Отдельной трудностью может быть необходимость коррекции пероральной противовоспалительной терапии с учетом возможного изменения фармакокинетики препаратов. Однако с выходом новых генно-инженерных биологических препаратов, а также широкой доступностью инъекционных форм метотрексата данная проблема становится менее актуальной для пациентов с ревматоидным и ПсА. Парентеральная терапия сопутствующего остеопороза также может быть более предпочтительной, учитывая и без того низкую биодоступность пероральных бисфосфонатов, а также их негативное влияние на верхние отделы ЖКТ [51].

## ЗАКЛЮЧЕНИЕ

Лечение ожирения является достаточно трудным процессом, требующим взаимодействия врача и пациента. В ряде случаев, несмотря на модификацию образа жизни и медикаментозную терапию, не удается достичь устойчивого снижения массы, что актуализирует проведение бариатрического вмешательства. Имеющиеся данные показывают возможность положительного влияния БХ не только на массу тела, но и на течение ряда ревматических заболеваний, что особенно важно ввиду затруднения снижения массы тела путем увеличения физической нагрузки у таких пациентов. Вместе с тем необходимо дальнейшее проведение проспективных исследований в данной области.

## ДОПОЛНИТЕЛЬНАЯ ИНФОРМАЦИЯ

Источники финансирования. Поисково-аналитическая работа выполнена в рамках фундаментальной научной тематики ФГБНУ НИИР им. В.А. Насоновой «Разработка персонализированной программы лечения рефрактерного ревматоидного артрита на основе изучения молекулярно-генетических и молекулярно-биологических предикторов. Создание и апробация регистра пациентов с ревматоидным артритом, резистентных к базисной противовоспалительной терапии» №1021051503137-7.

Конфликт интересов. Авторы декларируют отсутствие явных и потенциальных конфликтов интересов, связанных с содержанием настоящей статьи.

Участие авторов. Паневин Т.С. — концепция исследования, анализ данных, написание статьи, редакционная правка; Лила А.М. — концепция исследования, написание статьи, редакционная правка; Зоткин Е.Г. — анализ данных, редакционная правка. Все авторы одобрили финальную версию статьи перед публикацией, выразили согласие нести ответственность за все аспекты работы, подразумевающую надлежащее изучение и решение вопросов, связанных с точностью или добросовестностью любой части работы.
